# The Effect of Transformational Leadership and Remote Working on Employee Performance During COVID-19 Pandemic

**DOI:** 10.3389/fpsyg.2022.919631

**Published:** 2022-08-12

**Authors:** Meiryani, Nelviana, Yorick Koh, Gatot Soepriyanto, Mohammed Aljuaid, Fakhrul Hasan

**Affiliations:** ^1^Accounting Department, School of Accounting, Bina Nusantara University, Jakarta, Indonesia; ^2^Department of Health Administration, College of Business Administration, King Saud University, Riyadh, Saudi Arabia; ^3^Liverpool Hope University, Liverpool, United Kingdom

**Keywords:** COVID-19, transformational leadership, remote working, employee performance, leadership style

## Abstract

The COVID-19 outbreak has emphasized the importance of leadership style in achieving organizational performance. It also implies changes in administrative processes to remote working, impacting employee activities, and performance. Employee performance is one of the aspects that might influence a company’s success. If employees are productive and provide high-quality work, the company’s performance will increase. This study aims to analyze the effect of Transformational Leadership and Remote Working on Employee Performance during the COVID-19 pandemic. This study was conducted by performing a questionnaire distribution survey and acquiring 136 respondents. The research was conducted on a Jakarta area company that had implemented Remote Working. The number of samples in this study was determined using a simple random sampling procedure, in which the sampling was done at random without consideration for the population’s existing strata. This study uses a quantitative approach method based on factual data and research data in statistical figures related to concluding research problems. The source of data in this study is primary data that was directly obtained from sources without going through intermediaries. This study shows that Transformational Leadership has no significant impact on Employee Performance during the COVID-19 pandemic, and Remote Working has a significant impact on Employee Performance during the COVID-19 pandemic.

## Introduction

The spread of SARS-CoV-2, better known as COVID-19, was declared a pandemic by World Health Organizations (2020). The COVID-19 pandemic has forced many businesses to close and caused unprecedented trade disruptions in most industrial sectors. All organizational functions had to prioritize spending and delaying tasks that would not bring value to the environment (Donthu and Gustafsson, 2020). Due to COVID-19, most workers and companies have to do remote work even though they have little experience and are not ready to work remotely (Kniffin et al., 2021).

The COVID-19 pandemic has given us a valuable lesson. During this pandemic, governments worldwide imposed lockdown conditions, which resulted in a significant shift in daily activity from industrial and office locations to homes in a relatively short period (Kylili et al., 2020). Because team members work flexibly, remote work can lead to irregular work patterns. However, everyone has other commitments and priorities to prioritize, such as taking care of children or caring for relatives. Remote work can develop empathy in a team because it allows everyone to understand each other’s situation and avoid possible hatred and guilt over perceived inequalities (Phillips, 2020).

Many things changed when the COVID-19 pandemic struck, one of which was leadership practices. The PSBB (Large-Scale Social Restrictions) policy and the implementation of health protocols require leaders to adopt a new style of guarding the organization. Lenovo’s President in the Asia Pacific, Ken Wong, stated that COVID-19 was a catalyst for many companies to accelerate digital transformation. Almost all companies during the pandemic changed their communication patterns where they opened several communication channels, both digital and traditional. So leaders are required to master various communication channels, mainly digital media. However, the implementation of digital or remote work is also accompanied by an increasing workload. When it comes to working from home, there is work to be done, and 37 percent of the world’s workers feel that employers demand more from them. So company leaders also need to pay attention to the mental health problems of workers. During the COVID-19 period, some employees were laid off or lowered their salaries to keep the company afloat (Compass, 2021).

COVID-19 pandemic has a significant impact on the decline in employee performance and overall affects the achievement of company targets. According to Communication and National Motivator Aqua Dwipayana, many employees felt bored during the COVID-19 pandemic. With the change in the workplace, where everything is usually organized and done together in the office to working from their respective homes because they are prohibited from entering the office, communication and coordination become more challenging to implement. Moreover, this significantly affects employee results and performance (Solopos, 2021).

The transformational leadership style has begun to consider its usefulness in dealing with organizational changes. Empirical evidence shows that transformational leadership affects organizational effectiveness and employee performance (Keller, 1992). Research also shows that companies that provide a better work life balance through remote working pave the way for the workforce to be more productive because employees feel more motivated (Stevens, 2019). Graves and Karabayeva (2020) state that remote work gives employees more flexibility, more time availability because they are not spent on the road, and access to more excellent talent throughout the world, all of which help boost the average individual’s performance.

Based on this description, this study aims to analyze whether transformational leadership has a significant effect on the performance of company employees and whether remote working has a significant effect on the performance of company employees. The topic of this research has been carried out in several previous studies. Still, no research has been conducted simultaneously using transformational leadership and remote working variables. This study has a research gap in using transformational leadership and remote working as independent variables and testing their effect on the dependent variable, employee performance.

The expected contribution of this research is to provide empirical evidence of the effect of transformational leadership and remote working on employee performance. The results showed that transformational leadership had no significant effect on employee performance. It is hoped that it will be taken into consideration for leaders to change their leadership style to a more appropriate style, namely, a supportive leadership style, which directs employees to achieve organizational goals, not an authoritarian leadership style. The results of this study indicate that remote work has a significant effect on employee performance. So that it can be a reference for decision makers/companies who are still undecided in implementing remote work arrangements for their employees.

## Literature Review

*Employee performance* is the results and accomplishments obtained at work. “Performance” refers to sticking to a plan while aiming toward a specific outcome, even though performance evaluation is at the core of performance management (Cardy, 2004). Employee performance refers to an employee’s financial or non-financial outcome directly related to the organization’s performance and success (Anitha, 2013).

Transformational leadership is leadership that actively considers the needs and aspirations of followers. Leaders try to motivate subordinates and other stakeholders, to focus on the organization’s vision and mission, make group interests a priority, and motivate followers to go beyond personal interests (Raffo and Williams, 2018). Transformational leaders have high expectations for their followers and believe in their abilities. They inspire, empower, and stimulate followers to perform above and beyond their normal levels, and transformational leaders are also concerned with their followers’ personal needs and development (Bernard and Ronald, 2005). *Transformational leadership* is a process in which leaders and followers jointly enhance and develop their morality and motivation. Leaders who apply a transformational leadership style make their followers see that achieving goals are more than just their interests (Yukl, 2010). According to Bass and Riggio (2006), the transformational leadership dimension is divided into idealized influence, inspirational motivation, intellectual stimulation, and individualized consideration. The most critical dimension of transformational leadership is idealized influence, which inspires and motivates subordinates (emotionally) to put aside personal interests to attain collective goals (Sabaruddinsah and Asiah, 2022). Transformational leadership style can bring changes that will impact the emergence of employee motivation to make extra efforts in achieving the expected performance. So that having a transformational leader will improve the performance of company employees better.

This statement is supported by the research of Sabaruddinsah and Asiah (2022), which shows that transformational leadership has a positive and significant impact on employee performance, implying that the stronger the transformational leadership, the better the implementation resulting in increased employee performance. Jufrizen and Lubis (2020) also states that transformational leadership significantly affects employee performance. Another supporting research is the study of Faiza et al. (2019). According to the study, transformational leadership significantly impacts employee performance. Implementing transformational leadership will increase employee performance by enhancing work satisfaction among employees. At the same time, Masduki et al. (2020) showed different research results which stated that transformational leadership had no significant effect on employee performance. This could be due to the age factor of the employees, who mainly were mature, and the dominant employee working period was long, so the existence of transformational leadership had little effect on employee performance because they are relatively more independent.

According to the Aunthenticated U.S Government Information (2010), the term “telework” or “teleworking” refers to a work flexibility arrangement in which an employee performs his or her position’s duties and responsibilities, as well as other authorized activities, from a location other than the one from which the employee would usually work (Aunthenticated U.S Government Information, 2010). Remote working can be interpreted as a flexible work arrangement where employees work in different locations, far from the office. Employees do not have direct contact with existing co-workers but can communicate with co-workers using existing technology (Di Martino and Wirth, 1990). Problems stemming from remote working are numerous, ranging from travel restrictions and postponement of important meetings and events that will reduce employees’ ability and willingness to perform tasks. In addition, employees who work remotely due to COVID-19 have a higher chance of experiencing anxiety, frustration, and fatigue. It can affect their productivity and engagement and make the quality of employee performance results decrease and be prone to errors (Narayanamurthy and Tortorella, 2021). This statement is supported by research conducted by Kelvyn et al. (2021)and Khotimah et al. (2021), which states that remote working significantly affects employee performance. Another supporting research is the research of Narayanamurthy and Tortorella (2021), which shows the results of remote working COVID-19 has a direct impact on employee performance; this is believed because individual performance is not so vulnerable and does not rely on working conditions, relying more on individual factors such as adaptability and intrinsic motivation. Meanwhile, Suspahariati and Susilawati (2020) showed different research results that stated that remote working negatively impacted employee performance.

Compass (2021) during the COVID-19 period, some employees were laid off or lowered their salaries to keep the company afloat. This case causes many people to experience anxiety, frustration, and fatigue. In addition, according to Communication and National Motivator Aqua Dwipayana, many employees felt bored during the COVID-19 pandemic. This has a significant impact on the decline in employee performance and overall affects the achievement of company targets (Solopos, 2021). This phenomenon has a significant effect on employee results and performance. A supportive leadership style is essential in providing direction to employees, especially during the current COVID-19 pandemic, where transparency is essential. The leadership needed is leadership that can empower, guide, and direct employees. Leadership that can motivate employees is leadership that can foster the self-confidence of employees in carrying out their duties. This is supported by Bryans (2014) research showing that the better the leadership style in a company impact, the higher the company’s performance. This explains that the tendency to improve the quality of employee work is more influenced by corporate leadership style than the effect on career development. This can be seen from the factors that reflect the motivation of employees who do not prioritize work quality in career development. Employees assume that the decisions taken by the leadership are prioritized to be implemented so that this research contributes to the company as information and evaluates the performance of employees and company leaders.

### Hypothesis Development

#### Leadership Theory

The leader’s behavior that forms a continuity from autocratic to democratic nature is influenced by the intensity of the leader’s use of power and the use of freedom of followers.

1. Trait theory. The beginning of the emergence of this theory is that at that time people believed that leaders were born, not made. This theory is related to the great man theory, the difference between this theory is the talent that a person has. The Great Man theory emphasizes the talent of heredity that a leader has chromosomes (carriers of traits) from his parents as leaders. The trait theory is that leaders who have certain traits and people who pay attention to friendliness and build behavioral structures will look more effective (Robbins and Judge 2017, p. 252). Winardi (2000, p. 66) identifies the qualities that a leader must possess, including: intelligence, initiative, energy or stimulation, emotional maturity, persuasion, communicative skills, self-confidence, perceptiveness, creativity, and social participation. Davis (1972) formulated four general traits that have an influence on the success of an organization’s leadership, namely: (1) intelligence; (2) maturity and breadth of social relations; (3) self-motivation and achievement drive; and (4) attitudes of human relations. Gibson (1985) see the value of the trait-based approach in the statement “the view that leadership as a whole is situational so that no personal characteristics can predict leadership, seems to overemphasize the nature of the situation and underestimate the personal nature of leadership.”
2. Behavioral Theory. Around the 1940s, researchers began exploring the expression that the way a person acts determines the effectiveness of that person’s leadership. The researchers examined behavior and its impact on the performance and satisfaction of subordinates. But now there is a well-known behavioral theory in leadership.
3. Terry Theory. Leadership as an activity to influence people to work willingly for achieve common goals. According to Anoraga, leadership is ability to influence others, through good communication directly or indirectly with the intention of moving people so that with understanding, awareness, and pleasure willing to follow the will of the leader. A leader must able to set goals to be achieved by the organization or company, in this context a leader must be able to design the right tactics and strategy. The theory of types of leadership styles was developed by House et al. (2004), revealed that someone the leader uses a style situation-dependent leadership: (a) Directive Leadership. The leader gives specific advice to the group and strengthen basic rules; (b) Supportive Leadership. There is a good relationship between leader with group and show sensitivity to member needs; (c) Participatory Leadership. Leaders make decisions based on consultation with the group and share information with the group; and (4) Achievement-Oriented Leadership. The leader confronts the members on challenging goals and encourages high performance, while shows confidence in group ability. Successful employees are employees who are able to produce good performance on the basis of their leadership and leadership style. Leaders must be able to use a leadership style that is acceptable to employees so that employees can carry out their duties properly. If the given task can be done well, the employee’s performance can increase and the company’s goals can be achieved.
4. Trait Theory of Leadership. Adherents of the trait theory of leadership believe that a leader is born not created (Luthans, 2008). Successful leaders have characteristics that are innate, not made (Bateman and Snell, 2004, p. 371). This theory asserts that a person is born with certain innate characteristics that allow them in certain historical situations or periods to emerge as a leader. By using this theoretical approach, when explaining leadership success, the main concern is to identify what personality traits a successful leader possesses. Because this theory emphasizes the characteristics of the person, this theory is also known as the “great person” theory (Luthans, 2008). Although it has been criticized a lot, because it sees many weaknesses, such as the difficulty of seeing the relationship between trait characteristics and leader success, this theory is still alive and is practiced in many organizations. In its development, the use of the trait of personality characteristic has shifted toward job related skills. Besides being born with certain traits, for the purpose of effective management, a leader is also required to have technical, conceptual, and human skills (Blumler and Katz, 1974). Yukl (2010, p. 70), in detail, involves creativity, organization, persuasiveness, diplomacy & tactfulness, knowledge of the task, and the ability to speak well as skills needed by a leader. Daft (2003, p. 519) then also explains the following personal characteristics that a leader must possess as: (1) Physical characteristics, including: Energy (energy) and physical endurance (physical stamina); (2) Intelligence, including: intelligence/intelligence, cognitive ability (cognitive ability), knowledge (knowledge), judgment (judgment), decisiveness (decisiveness), and fluency in language (fluency of speech); (3) Personality, including: self-confidence, honesty and integrity, enthusiasm, desire to lead, and independence. Alertness, creativity, emotional balance, and independent control (nonconformity); (4) Social characteristics include the ability to socialize (sociability), social skills (interpersonal skills), cooperation (cooperativeness), the ability to establish cooperation (ability to enlist cooperation), tactical, and diplomatic (tact and diplomacy); (5) Work related characteristics, including: drive to achievement, drive to excel, meticulous, careful in achieving goals, conscientiousness in pursuit of goals obstacle, persistent, and tenacity (tenacity); and (6) Social background, including: educational background, and mobility.
5. Contingency theory of leadership. Contingency leadership theory can be explained through a situational approach and path-goal model. In the situational approach, a leader is seen as a product of time and situation (Luthans, 2008, p. 414). People who have certain qualities or traits because of a situation will emerge as a leader. In another sense, the situational approach uses an assumption that there are several situational variables that can affect leadership roles, skills, behaviors, and follower’s performance and satisfaction. This situational variable is known as an attempt to perfect the previous trait theory which was seen as inadequate as a complete leadership theory (Luthans, 1995, p. 349). One of the initiators of the contingency leadership theory is Fred Fiedler, who is known as the recognized situation-based or contingency theory for leadership effectiveness. With this contingency model of leadership effectiveness, Fiedler explains the relationship between leadership style (style of leadership) and favorable situations (favorableness of the situation). The leadership style consists of task directed and human oriented (democratic) while for the favorableness of the situation, Fiedler divides it into the following three dimensions: (1) The leader-member relationship is the most critical variable in determining the desired situations; (2) The degree of task structure is the second very important variable in entering the desired situation; and (3) The leader’s position power obtained through formal authority, which is the third most critical variable or dimension. A leader is said to be in a favorable situation if all the dimensions stated above get the highest score. In other words, a leader is generally said to be accepted by subordinates (dimension I), all work is structured and supported by clear job descriptions (dimension II), and there are situations where between the authority and power that is formally carried by a leader (dimension III). Another situational leadership theory (SLT) model was developed by Hersey and Blanchard which divides specific leadership styles into four groups (Ivancevich, 2011, p. 454), namely: (1) Telling (high task-low relationship), leadership defines roles and tells people what (what), how (how), when (when), and where (where) to carry out various tasks. As a result, employees/subordinates are unable and do not want to take responsibility for doing something, followers are incompetent or insecure; (2) Selling (high task-hight relationship). Leaders provide behavioral direction and support. Employees/subordinates do not have the ability but are willing to do the work required by work tasks. Workers have motivation but do not have the appropriate skills; (3) Participating (low task-high relationship), the leader and his followers share in making decisions: the main role of the leader is to facilitate and communicate. Employees/subordinates have the ability but do not want to do what the leader wants. Followers are competent but do not want to do anything; and (4) Delegating (low task-low relationship), the leader provides little direction or support. Employees/subordinates have the ability and desire to do what is asked of him.
6. Path-Goal Theory of Leadership. The path-goal model is another approach to the contingency leadership theory proposed by Robert House (Snale and Bateman 2004, p. 381). Path-goal leadership theory was developed from a contingency approach derived from the expectancy framework of motivation theory (Luthans, 2008, p. 420). The purpose of this theory is to explain how a leader’s style/behavior affects the motivation, satisfaction, and performance of his subordinates. According to House (Luthans, 2008, p. 421), there are four types or styles/behaviors that can influence effective leadership, namely: (1) Directive leadership: the leadership style is the same as the authoritarian leader style referred by Lippit & White. That is, subordinates know exactly what the leader expects of them, and the leader gives specific directions on how employees should complete tasks. In this case, the subordinates are not involved at all by the leader; (2) Supportive leadership: The leader pays attention to the needs of his followers and is friendly; (3) Participative leadership, the leader consults with group members and uses group member suggestions before the leader makes a decision (decisions are still made by the leader); and (4) Achievement-oriented leadership. Leaders set challenging goals for their followers, giving followers confidence that these goals can be achieved to the best of their ability. Based on the description above, the hypotheses proposed in this study are as:

*H1:* Transformational Leadership significantly affects employee performance.

*H2:* Remote Working significantly affects employee performance.

## Materials and Methods

**Figure 1:**
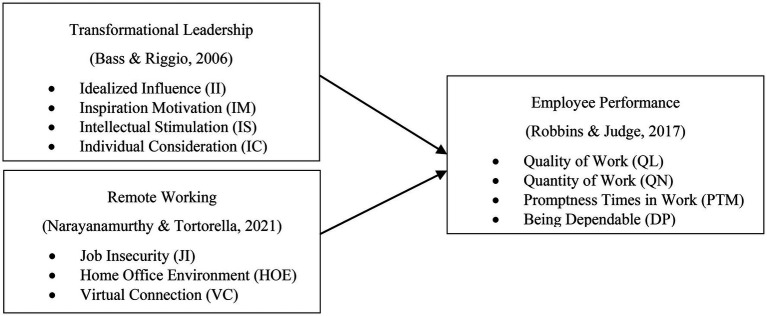
Conceptual framework.

The conceptual framework of this study is given in Figure 1.

### Population and Sample

The population is a generalization area made up of things or people that have specified numbers and qualities that researchers have identified and conclusions drawn from Sugiyono (2011, p. 80). The population in this study were 200 employees at a Jakarta company that implemented remote working during the pandemic. At the same time, the sample was representative of the population’s size and characteristics Sugiyono (2011, p. 81). The sampling method in this study is Simple Random Sampling in which the sample is taken randomly without regard to the existing strata in the population. This method can be used if the population in the study is not too large. The method used to determine the sample size is Slovin’s formula. Based on the Slovin’s formula, the estimation of sample that should be obtained in this study was 133 sample. And the sample obtained in this study amounted to 136 respondents as variance shown in Table 1.


$$n=\frac{N}{1+Ne^{2}}$$

**Table 1:** Demographic information.

Controls		Variance	
Age	<20 Years	7	(5.15%)
	20–30 Years	125	(91.91%)
	30–40 Years	4	(2.94%)
Working Period	< 2 Years	107	(78.68%)
	2–5 Years	25	(18.38%)
	5–7 Years	1	(0.73%)
	7–10 Years	3	(2.21%)
Position	Entry Level	117	(86.03%)
	Middle Level	16	(11.76%)
	High Level	3	(2.21%)

n = Total Sample, N = Total Population, and e = Margin Error.


$$n=\frac{200}{1+200.0,05^{2}}=133,33$$

### Measures

The research object used in this study consisted of the Independent Variable and the Dependent Variable as shows in Table 2. The first independent variable is transformational leadership as measured by four indicators, namely, idealized influence, inspirational motivation, intellectual stimulation, and individualized consideration (Bass and Riggio, 2006). The second independent variable is remote working which is measured by three indicators, namely, job insecurity, home office environment, and virtual connection (Narayanamurthy and Tortorella, 2021). While the dependent variable in this study is employee performance measured through four indicators, namely, quality of work, the quantity of work, promptness time in work, and being dependable (Robbins and Judge, 2017). The data in this study were collected using a questionnaire survey method distributed to respondents. With a total of 22 questions using a 5-point Likert Scale varying from 1 to 5, score 1 means strongly disagree; 2 means disagree; 3 means neutral; 4 means agree; and 5 means strongly agree. The questionnaire quality has been test using validity and reliability in Table 3 and shows that the data obtained in this study is valid and reliable to use. The reason the author chooses this analytical techniques is because it refers to the research of Phillips (2020); Narayanamurthy and Tortorella (2021); Sabaruddinsah and Asiah (2022) where their method fits within the scope of our study.

**Table 2:** Operationalization.

Scale	Dimension	Indicator	Score	Scale
Transformational Leadership	Idealized Influence (II)	II1	1 = Strongly Disagree	Likert Scale
		II2	2 = Disagree	
	Inspiration Motivation (IM)	IM1	3 = Neutral	
		IM2	4 = Agree	
	Intellectual Stimulation (IS)	IS1	5 = Strongly Agree	
		IS2		
	Individual Consideration (IC)	IC1		
		IC2		
Remote Working	Job Insecurity (JI)	JI1	1 = Strongly Disagree	Likert Scale
		JI2	2 = Disagree	
	Home Office Environment (HOE)	HOE1	3 = Neutral	
		HOE2	4 = Agree	
	Virtual Connection (VC)	VC1	5 = Strongly Agree	
		VC2		
Employee Performance	Quality of Work (QL)	QL1	1 = Strongly Disagree	Likert Scale
		QL2	2 = Disagree	
	Quantity of Work (QN)	QN1	3 = Neutral	
		QN2	4 = Agree	
	Promptness Times in Work (PTM)	PTM1	5 = Strongly Agree	
		PTM2		
	Being Dependable (DP)	DP1		
		DP2		

**Table 3:** Validity and reliability test.

Scale	Item	Corrected item-total correlation	Cronbach’s alpha if item deleted	Cronbach’s alpha
Transformational Leadership				0.871
	II1	0.469	0.863	
	II2	0.485	0.863	
	IM1	0.386	0.865	
	IM2	0.393	0.865	
	IS1	0.619	0.858	
	IS2	0.476	0.862	
	IC1	0.474	0.862	
	IC2	0.522	0.861	
Remote Working				0.712
	JI1	0.228	0.874	
	JI2	0.355	0.867	
	HOE1	0.460	0.863	
	HOE2	0.635	0.856	
	VC1	0.500	0.861	
	VC2	0.411	0.864	
Employee Performance				0.842
	QL1	0.537	0.861	
	QL2	0.552	0.860	
	QN1	0.563	0.860	
	QN2	0.493	0.862	
	PTM1	0.523	0.861	
	PTM2	0.371	0.866	
	DP1	0.330	0.868	
	DP2	0.462	0.863	

*Source: Output SPSS 25.*

### Data Analysis

The data in this study will be tested and analyzed through the SPSS 25 application. The tests that will be carried out are Validity & Reliability Test to determine the questionnaire quality, Collinearity Test to determine whether there was collinearity between independent variables, and Hypothesis Testing to answer the problem in research. The hypothesis will be test using Partial T-Test that is used to determine whether each independent variable individually affects the dependent variable. T-test was conducted with a significance level of 0.05 and T_table_ of 1.978. If the significance value is <0.05 and T_count_ > T_table_, then the hypothesis is accepted. This research is a multiple linear regression method. Testing the coefficient of determination is carried out with the intention of measuring the ability of the model to explain how the influence of the independent variables (simultaneously) affects the dependent variable which can be indicated by the adjusted R-Squared value (Ghozali, 2016). The coefficient of determination shows the extent to which the contribution of the independent variables in the regression model is able to explain the variation of the dependent variable. The coefficient of determination can be seen through the value of R-square (R2) in the Model Summary table. Adjusted R Square is the value of R Square that has been adjusted, this value is always smaller than R Square and this number can have a negative value. According to Santoso (2001) that for regression with more than two independent variables, Adjusted R2 is used as the coefficient of determination. R squared is a number that ranges from 0 to 1 which indicates the magnitude of the combination of independent variables which together affect the value of the dependent variable. The closer to number one, the model issued by the regression will be better. The coefficient of determination can be seen through the value of R-square (R2) in the Model Summary table.

### Variable Operationalization

This research consists of two independent variables, namely, transformational leadership (X1) and Remote Working (X2) and one independent variable, namely, Employee Performance. Transformational leadership is measured by the dimensions of idealized influence (II), inspiration Motivation (IM), Intellectual Stimulation (IS), and Individual Consideration (IC). The Remote Working variable is measured by the dimensions of Job Insecurity (JI), Home Office Environment (HOE), and Virtual Connection (VC). Employee Performance variables are measured by the dimensions of Quality of Work (QL), Quantity of Work (QN), Promptness Times in Work (PTM), and Being Dependable (DP). Where the three variables in this study are reflective variables. Because the indicators in the three variables are the consequences of the construct, the construct describes the indicators, if the construct assessment changes then all indicators will change and indicators can be exchanged equally. Operationalization variable table can be seen in the appendix, namely, in Table 2 Operationalization.

## Results and Discussion

### Validity and Reliability Test

Validity test is used to determine whether a measuring instrument has performed its measuring function. Validity test was carried out with a significance level of 0.05 and R_table_ of 0.168. If the value of R_count_ > R_table_, then the data obtained is declared valid. Based on the test results in Table 3, each indicator measuring the variables of Transformational Leadership (TL), Remote Working (RW), and Employee Performance (EP) has T_count_ > T_table_ (0.168) so all measuring indicators in this study are declared valid and feasible to use. Reliability test is a test used to measure the accuracy and reliability of a measuring instrument. Data are declared reliable if the value of Cronbach’s Alpha is greater than 0.60. Based on the test results in Table 3, each variable Transformational Leadership (TL), Remote Working (RW), and Employee Performance (EP) has a Cronbach’s Alpha value >0.60 so all measuring variables in this study are stated to be reliable and feasible to use.

### Descriptive Statistic

Based on the descriptive statistical tests presented in Table 4, the data used in this study are 136 sample. The result show that Transformational Leadership variable has the minimum and maximum values of 19 and 40, with mean value of 35.38 and the standard deviation is 4.151. The Remote Working variable has shown the minimum and maximum values of 15 and 30, with mean value of 24.11 and the standard deviation is 3.928. The Employee Performance variable has shown the minimum and maximum values of 24 and 40, with mean value of 33.26 and the standard deviation is 4.389.

**Table 4:** Descriptive statistic.

	N	Minimum	Maximum	Mean	Std. deviation
Transformational Leadership	136	19	40	35.38	4.151
Remote Working	136	15	30	24.11	3.928
Employee Performance	136	24	40	33.26	4.389
Valid N (listwise)	136				

### Normality Test

**Figure 2:**
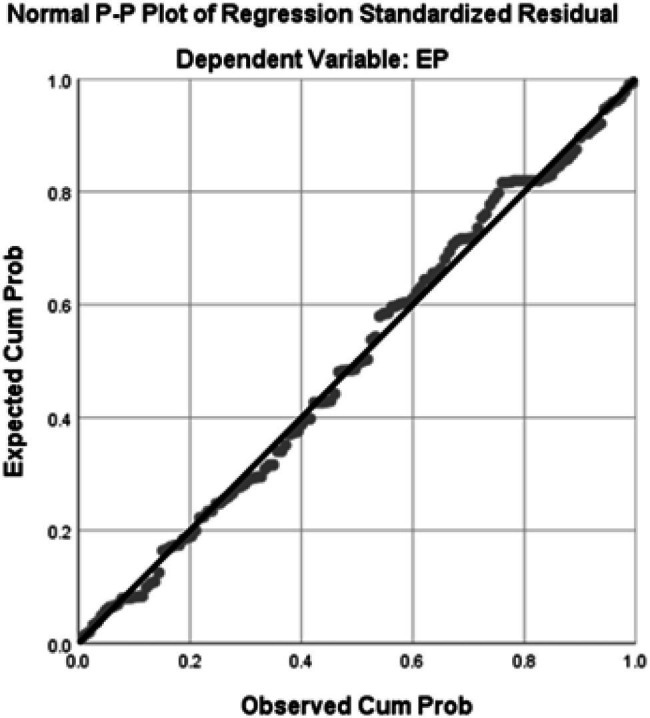
Normality test.

The normality test of the data was carried out by showing whether the dependent variable and the independent variable in this study were normally distributed. The results of the normality test in Figure 2 show that the research variables are normally distributed, which is indicated by the data that spreads around the diagonal line and follows the diagonal line.

### Coefficient of Determination Test (Adjusted R2) Result

With a value ranging from 0 to 1, the coefficient of determination can indicate how effective the independent variable is at explaining the variance of the dependent variable. The closer the value is to 1, the more effective the independent variable is at explaining the variance of the dependent variable. According to the test results in Table 5, the Adjusted R2 value is 0.188, indicating that the independent variables Transformational Leadership (TL) and Remote Working (RW) influence the dependent variable Employee Performance (EP) by 18.8% and other variables outside the research model by 81.2%, respectively.

**Table 5:** Coefficient of determination test (adjusted R2) result.

Model	*R*	*R* Square	Adjusted *R* square	Std. error of the estimate
1	0.448^a^	0.200	0.188	3.954

^a^Predictors: (Constant), Remote Working, Transformational Leadership; ^b^Dependent Variable: Employee Performance.

### Collinearity Test

Multicollinearity test was conducted to ascertain whether there was intercorrelation or collinearity between independent variables in a regression model. If the VIF value is <10 or the Tolerance value is >0.01, it is stated that there is no multicollinearity. Based on Table 6, the VIF value is 1.193 < 10 and the Tolerance value is 0.838 > 0.01 so it can be concluded that multicollinearity is not detected.

**Table 6:** Collinearity diagnostic result.

Model		Unstandardized coefficients		Standardized coefficients	*t*	Sig.	Collinearity statistics	
		B	Std. error	Beta			Tolerance	VIF
1	(Constant)	18.102	3.088		5.861	0.000		
	TL	0.142	0.090	0.134	1.581	0.116	0.838	1.193
	RW	0.421	0.095	0.377	4.449	0.000	0.838	1.193

^a^Dependent Variable: Employee Performance.

### Simultaneous F-Test Result

The F test is known as the Simultaneous Test or the Model Test/Anova Test, which is a test to see how the effect of all the independent variables together on the dependent variable. Or to test whether the regression model that we make is good/significant or not good/non-significant. In this article, it is explained about the F-Test and T-Test in research. If the model is significant then the model can be used for prediction/forecasting, otherwise if it is not/significant then the regression model cannot be used for forecasting. The F test can be done by comparing F arithmetic with Table F: F Table in Excel, if F count > from F table (Ho is rejected and Ha is accepted) then the model is significant or can be seen in the significance column on ANOVA (Processed with SPSS, Use Test Regression with Enter/Full Model Method). The model is significant as long as the significance column (%) < Alpha (readiness to err type 1, which is what the researcher decides, social sciences usually have at most an alpha of 10% or 5% or 1%). And vice versa if F count < F table, then the model is not significant, it is also indicated that the value of the significance column (%) will be greater than alpha. Simultaneous Test F or ANOVA Test is a test used to test the significance of all independent variables as a whole on the dependent variable. The significance value used in the F test is 0.05 with a F_table_ value of 3.06. From the results of the simultaneous F test shown in Table 7, the *F* value of 16.67 is obtained so that F_count_ (16.67) > F_table_ (3.06), which indicates that the independent variables of Transformational Leadership (TL) and Remote Working (RW) mutually affect the dependent variable is Employee Performance (EP).

**Table 7:** Simultaneous *F*-test result.

Model		Sum of squares	df	Mean square	*F*	Sig.
1	Regression	521.123	2	260.561	16.670	.000^b^
	Residual	2078.870	133	15.631		
	Total	2599.993	135			

^a^Dependent Variable: Employee Performance; ^b^Predictors: (Constant), Remote Working, Transformational Leadership.

### Partial Regression Coefficient Test (t-Test) Result

The t-test is one of the statistical tests to test the truth of the hypothesis proposed by the researcher in differentiating the average in the two populations. Parametric statistical tests have several types of tests that are used to obtain conclusions about the population from the samples taken. The t-test is known as the partial test, which is to test how the influence of each independent variable individually on the dependent variable. This test can be done by comparing t count with t table or by looking at the significance column for each t count, the t test process is identical to the F test (see SPSS calculation on Coefficient Regression Full Model/Enter). Or can be replaced with Stepwise Test method. Partial T-Test is used to test whether each independent variable individually affects the dependent variable. T-test was conducted with a significance level of 0.05 and T_table_ of 1.978. If the significance value is <0.05 and T_count_ > T_table_, then the hypothesis is accepted.

#### The Effect of Transformational Leadership on Employee Performance

Based on the test results in Table 8, the independent variable Transformational Leadership (TL) has a value of T_count_ (1.581) < T_table_ (1.978) and value of *p* (0.116) > 0.05, so H1 “Transformational Leadership has a significant effect on employee performance” is rejected. The purpose of this study was to see how transformational leadership affected employee performance as measured by idealized influence, inspirational motivation, intellectual stimulation, and individualized consideration. According to Nothouse (2013), transformational leadership is social leadership that is more concerned with other people and the common good.

**Table 8:** Partial regression coefficient test (*t*-test) result.

Model		Unstandardized coefficients		Standardized coefficients	*t*	Sig.
		*B*	Std. error	Beta		
1	(Constant)	18.102	3.088		5.861	0.000
	TL	0.142	0.090	0.134	1.581	0.116
	RW	0.421	0.095	0.377	4.449	0.000

^a^Dependent Variable: Employee Performance.

Leadership style directly has a significant influence on employee performance. This shows that when a given leadership style is good, it will have an impact on better employee performance. The results of this study are in accordance with the theory put forward by Siagian which reveals that leadership is a person’s ability to influence others, in such a way that other people want to do the leader’s will even though he personally dislikes it (Siagian, 2002). This shows that the actions of a leader are the rudder for a company in going through the ups and downs of the company’s conditions, and leadership style is a leader’s technique in treating employees and employee motivation. This study also supports the theory of performance proposed by Mathis and Jackson (2006) explaining that performance is basically what the employee does and does not do. This research is supported by previous research conducted by Bryans (2014) which found that leadership style has a significant effect on employee performance.

For the sake of success in carrying out the vision and mission to achieve company goals, it is necessary to improve the quality of human resources, one of which is by having a leader who can be an example for his followers (Jufrizen and Lubis, 2020). Transformational leadership is a process in which leaders and followers jointly enhance and develop their morality and motivation. Leaders who apply a transformational leadership style make their followers see that achieving goals are more than just their personal interests (Yukl, 2010). Transformational leaders do not only look at the company side, but also see it from the employee side, so it is believed that employees will be encouraged to work even better to achieve common interests. Indirectly, employee performance will undoubtedly increase.

The leadership style that needs to be maintained in achieving company performance such as in terms of decision making and the need to improve aspects where the leadership must pay attention to the interests of employees and the interests of the company.

The results of this study are supported by the theory put forward by Amirullah (2015, p. 167), leadership is a person who has the authority to give assignments, has the ability to persuade or influence others through good relationship patterns in order to achieve predetermined goals, and is supported by theory as suggested by Kartika (2014), the characteristics of a democratic leadership style are as follows: First, decisions and policies are made jointly between leaders and subordinates. Second, communication takes place reciprocally, both between leaders and subordinates as well as fellow subordinates and third, there are many opportunities for subordinates to convey suggestions, considerations, or opinions. The results of this study support the results of Dionysius’ (2014) research. The results show that employee performance that needs to be maintained is in the aspect where every job given to employees is done carefully. Employee performance that needs to be improved is the aspect where employees must be able to adapt to the work environment.

The transformational leader inspires followers by making them more aware of the importance of task results, encouraging them to put the needs of the company or team ahead of their own, and activating their higher-order demands. He challenges his followers to think critically and explore fresh approaches to their employment, resulting in intellectual stimulation (Bass and Avolio, 1994). As a result, their level of performance, contentment, and dedication to the organization’s goals has increased (Podsakoff et al., 1996).

However, another opinion states that transformational leadership does not affect employee performance. This can happen because of the age factor of the employees, the majority of employees are mature, and the working period is long, so the existence of transformational leadership does not have much influence on employee performance because they are already relatively more independent (Masduki et al., 2020).

From the results of hypothesis testing that has been carried out, it can be concluded that H1 is rejected. This is in line with research conducted by Masduki et al. (2020), which states that Transformational Leadership has no significant effect on employee performance. Purwanto et al. (2019) conducted a study on lecturers at a private university in the Tangerang area and concluded that transformation had no significant effect on employee performance. Sudiantha and Armanu Troena (2017) also state that transformational leadership has no significant impact on employee performance. This shows that the leader lacks the ability to motivate and transfer expectations and goals to employees. Leaders must utilize understandable and straightforward methods to elicit more valuable contributions and an excellent performance from their employees. Furthermore, leaders must have a strong bond with their staff and maintain open lines of contact with them, as well as provide advice and care.

#### The Effect of Remote Working on Employee Performance

Based on the test results in Table 8, the independent variable Remote Working (RW) has a value of T_count_ (4.449) > T_table_ (1.978) and value of *p* (0.000) < 0.05, so H2 “Remote Working has a significant effect on employee performance” is accepted. This research was conducted to determine the effect of Remote Working on Employee Performance as measured by Job Insecurity, Home Office Environment, and Virtual Connection as indicators. According to Martiono and Wirth (1990), remote working is a flexible work arrangement where workers operate in different locations, often far away. Workers do not have direct contact with co-workers outside of the workplace, but they can interact with them using existing technologies. Remote working created based on critical national situations and conditions. The application of work from home has four goals, namely: reducing crowds, preventing indirect transmission of COVID-19, reducing employees work activities outside the home, and maintain the health of employees and their families. Work activities in outside the home can pose a high risk.

There are some positive impacts of implementing remote working. Because of the implementation of remote working, individual performance is less vulnerable and does not rely on working conditions, relying more on individual factors such as adaptability and intrinsic motivation. In addition, employees can also spend more time with their families and can do other more urgent things. This is believed to increase the morale and emotions of employees so that employees will certainly be more enthusiastic about doing their jobs without the burden of thoughts from factors outside of work.

According to research done by Stanford University, productivity among work from home employees increased by 13%, with the majority of that gain attributed to working longer hours. Virtual workers are more productive because they can work without interruptions, allowing them to make the most of their time. Virtual labor, on average, results in increased satisfaction, decreased absenteeism, and higher retention (Flores, 2019).

Running remote work requires smooth Internet access so that the work process will be more efficient. However, not all companies are able or ready to carry out remote working. This is because Internet access tends to be expensive, and not all companies can facilitate all employees who have different residences to get Internet access. In addition, the government has not been able to facilitate Internet access to all regions. Apart from smooth Internet access, there are many limitations due to remote working. This is because not all work processes can be done online. During the COVID-19 pandemic, many agendas that were usually carried out on site had to be changed to online. This is undoubtedly a challenge for every company to maximize the plan.

From the results of hypothesis testing that has been carried out, it can be concluded that H2 is accepted. This is in line with research conducted by Narayanamurthy and Tortorella (2021), which examined the effect of COVID-19 on employee performance during the COVID-19 pandemic and showed that remote working could improve the quality and performance of employees. Hutaluju (2021) states that there is a positive relationship between work from home and work motivation and positive implications for job performance. They are supported by Kelvyn et al. (2021), which prove that work motivation and job satisfaction positively affect work performance. The job satisfaction factor has a significant influence on work performance. If job satisfaction is not achieved, work stress will be generated. Thus, employee performance will be poor, and productivity can also be negatively affected.

## Conclusion

Based on the results of data analysis in this study, it can be concluded that transformational leadership has no significant effect on employee performance. Leadership style has a positive effect on employee performance. If the leadership style of employees is further improved, then employee performance will increase, and vice versa if the leadership given to employees is low, employee performance will decrease. This shows that if the leadership given to employees is good, then employee job satisfaction will be created so that employees will be more enthusiastic at work. And vice versa if leadership given to employees is low, employee job satisfaction will not create in employees. Therefore, the transformational leadership style does not always increase the performance of employees. It can also be because the transformational leadership carried out by leaders in the company has not been implemented well toward the employees, so it does not improve their performance. The results of Nurlia’s research (2017) show that a person’s leadership style is influential and becomes a determining factor for increasing and decreasing the performance of subordinates; therefore, it appears that every company needs a more effective leadership style because apart from depending on the ability of employees to carry out work, it is also clear that leadership is good. More effective and the role of leadership is very influential because the success of the organization can be determined of the leadership style applied to the organization to achieve the goals of the organization.

Remote working has a significant effect on employee performance. Remote working should be improved effectiveness and efficiency of the system and look for appropriate mechanisms to Employee performance increased. These findings indicate that if the implementation of remote working can be carried out effectively in accordance with established procedures and not become a means for employees to be lazy, then employee performance will increase. The results of the study show that supervision less stringent so far has affected the performance of employees; therefore, supervision must be more stringent by conducting audits in the office, frequently conducting zoom meetings, and giving strict sanctions to employees who violate the regulations. The company supports the cost of the Internet network where remote working depends on the data network and distance. If the data network encounters problems, then the work results of employees experience obstacles such as hampered communication systems and hampered work processes. Therefore, a friendly remote working system applied in a company will further increase employee performance because remote working allows employees to work on a more flexible schedule and comfortable workplace, allowing work to be completed more efficiently. Researchers suggest that companies should still apply remote working arrangement even though the pandemic period is over in the future. It is hoped that further research can be developed using variables other than working from home and work stress such as work environment variables in previous research resulted in the conclusion of an influence on employee performance. Research results can be used as a reference or knowledge for researchers, academics, practitioners, and contribute to company policies in improving employee performance.

This study has a few limitations. Firstly, because the sample used is only the dominant community that works in one area, it cannot reflect the overall condition. So, the researcher suggests taking a sample with a broader area for future research. However, the total number of 136 samples has been estimated using Slovin’s Formula, so the sample size in this study is suitable according to the academic concept. Second, the employee performance variable was taken through a self-report of employee performance so that the results would be less objective. Future research should use a third party or company evaluation for the employee performance measurement. Last, this study argued that transformational leadership could increase the performance of employees. However, other leadership styles might be more appropriate for increasing employee performance. Future research can add other leadership styles (e.g., Participative Leadership and Transactional Leadership) that may impact employee performance too.

## Data Availability Statement

The original contributions presented in the study are included in the article/supplementary material, further inquiries can be directed to the corresponding author.

## Ethics Statement

Ethical review and approval was not required for the study on human participants in accordance with the local legislation and institutional requirements. Written informed consent from the patients/ participants or patients/participants legal guardian/next of kin was not required to participate in this study in accordance with the national legislation and the institutional requirements.

## Author Contributions

Meiryani and Nelviana contributed to reagents, materials, and analysis tools or data, conceived and designed the exploration, and wrote the paper. GS, YK, and Meiryani performed the experiments, conceived and designed the exploration, analyzed and interpreted the data, and wrote the paper. MA and FH contributed to funding, validation data, review, project administration, and editing final. All authors contributed to the article and approved the submitted version.

## Funding

The authors would like to extend their appreciation to King Saud University for funding this work through the Researcher Supporting Project (RSP2022R481), King Saud University, Riyadh, Saudi Arabia.

## Conflict of Interest

The authors declare that the research was conducted in the absence of any commercial or financial relationships that could be construed as a potential conflict of interest.

## Publisher’s Note

All claims expressed in this article are solely those of the authors and do not necessarily represent those of their affiliated organizations, or those of the publisher, the editors and the reviewers. Any product that may be evaluated in this article, or claim that may be made by its manufacturer, is not guaranteed or endorsed by the publisher.
